# Breast implant-associated anaplastic large cell lymphoma in a Li-FRAUMENI patient: a case report

**DOI:** 10.1186/s13000-018-0688-x

**Published:** 2018-01-25

**Authors:** Ricardo Garcia Pastorello, Felipe D’Almeida Costa, Cynthia A. B. T. Osório, Fabiana B. A. Makdissi, Stephania Martins Bezerra, Marina de Brot, Antonio Hugo J. F. M. Campos, Fernando Augusto Soares, José Vassallo

**Affiliations:** 1 Department of Anatomic Pathology, A.C. Camargo Cancer Center, 211 Professor Antônio Prudente Street, Sao Paulo, Zip code 01509-900 Brazil; 2 Department of Breast Surgery, A.C. Camargo Cancer Center, Sao Paulo, Brazil; 3 Department of Breast Surgery, A.C. Camargo Cancer Center, University of São Paulo, Sao Paulo, Brazil

**Keywords:** Lymphoma, Anaplastic large cell lymphoma, Anaplastic lymphoma kinase (ALK), Li-FRAUMENI syndrome, Breast implant

## Abstract

**Background:**

Breast implant-associated anaplastic large cell lymphoma (BIA-ALCL) is a rare malignancy, recently recognized as a provisional entity by the World Health Organization. Although increasing data have been published on this entity in recent years, a great number of patients and health professionals remain unaware of this diagnosis.

**Case presentation:**

We herein report the case of a 56-year-old female with Li-FRAUMENI syndrome who presented with late right-sided recurrent breast swelling after prophylactic adenomastectomy with implant reconstruction. Imaging scans revealed an heterogeneous mass adjacent to the implant fibrous capsule. A biopsy of the lesion rendered the diagnosis of a BIA-ALCL.

**Conclusions:**

This case presents similarities with previous reports, but also some particularities, which should be stressed in order to make the diagnosis the earliest possible. The most distinct feature is that this is the second report of BIA-ALCL arising in the setting of Li-FRAUMENI syndrome.

## Background

Primary breast lymphomas are rare malignancies, mostly of B-cell origin, estimated to represent around 0.5% of all primary breast neoplasms [1]. ALK-negative anaplastic large cell lymphomas (ALCL, ALK-) are CD30+ T-cell neoplasms, which lack anaplastic large cell lymphoma kinase (ALK) rearrangement and corresponding protein [2]. Since the publication by Keech and Creech in 1997 [3], an increasing number of cases on the association of ALCL, ALK- with breast implants have appeared, in patients with both aesthetic and reconstructive indications. In 2016, the World Health Organization (WHO) provisionally added breast implant-associated anaplastic large cell lymphoma (BIA-ALCL) as a newly recognized entity [2].

The incidence and prevalence of BIA-ALCL is extremely low. According to a recent epidemiological study by Doren et al. [4], the incidence of cases in textured breast implants was around 2 per 1 million person/years in the U.S. In the UK experience, the mean age at diagnosis was 52 years, with the mean time from implant surgery to lymphoma diagnosis ranging from 3 to 16 years [5].

In Brazil, only one publication on breast lymphoma included a case diagnosed as BIA-ALCL [6]. As this diagnosis may be obscured by the much more common granulomatous reaction to silicone, a new case is presented and discussed herein.

## Case presentation

A 56-year-old female with a family history of multiple malignancies was diagnosed with Paget disease of the nipple. She underwent modified left radical mastectomy, followed by prophylactic contralateral adenomastectomy and bilateral reconstruction with silicone implant. Microscopic evaluation of the surgical specimen revealed a left-sided microinvasive carcinoma in the background of high grade ductal carcinoma in situ. No further treatment was prescribed at that time. Few months later, after clinical evaluation and genetic testing, the patient was diagnosed with Li-FRAUMENI syndrome (LFS).

Seven years later, in the follow-up of the breast reconstruction, the patient came to consultation reporting right-sided recurrent breast swelling for the past 18 months. In the meantime, she had already been submitted to imaging studies, which revealed fluid collections adjacent to the implant, suggestive of inflammatory process, followed by fine needle aspiration which showed no signs of malignancy.

**Fig. 1:**
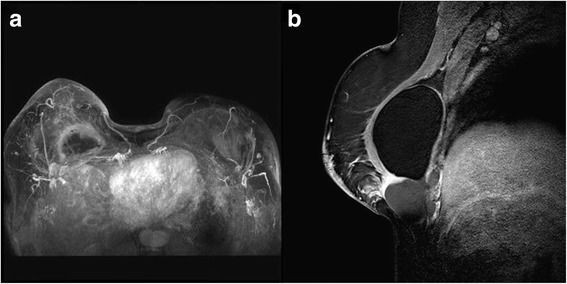
Magnetic resonance imaging showing a mass in the lower inner quadrant of the right breast with rim contrast-enhancement abutting the implant surface. **a** Sagittal fat suppressed contrast-enhanced T1-weighted image. **b** Maximum intensity projection image

The patient was then submitted to breast magnetic resonance imaging scan (MRI) (Fig. 1), which showed a 6 cm heterogeneous mass with a contrast peripheral enhancement, adjacent to the implant fibrous capsule in the right breast. This was associated with enlarged lymph nodes of the ipsilateral axillary and internal mammary chains. The lesion was biopsied.

### Pathologic findings

Biopsy fragments were 10% buffered neutral formalin fixed and paraffin embedded. Routine staining with hematoxylin and eosin (H&E) was performed, and additional sections were submitted to immunohistochemical phenotyping. The antibody panel included markers for cytokeratins (AE1/AE3), epithelial membrane antigen (EMA, E-29), CD45/CLA (RP2/18), CD20 (L26), PAX5 (SP34) CD2 (MRQ-11), CD3(2GV6), CD4(SP35), CD5 (SP19), CD7 (CBC37), CD8 (SP57), CD30 (Ber-H2), CD68 (KP-1), ALK (ALK01), TIA1 (C-20) and Ki-67 (30–9). CD2 was provided by Cell Marque; CD7 by Dako; TIA1 by Santa Cruz. All other markers and the automated system were provided by Ventana-Benchmark.

**Fig. 2:**
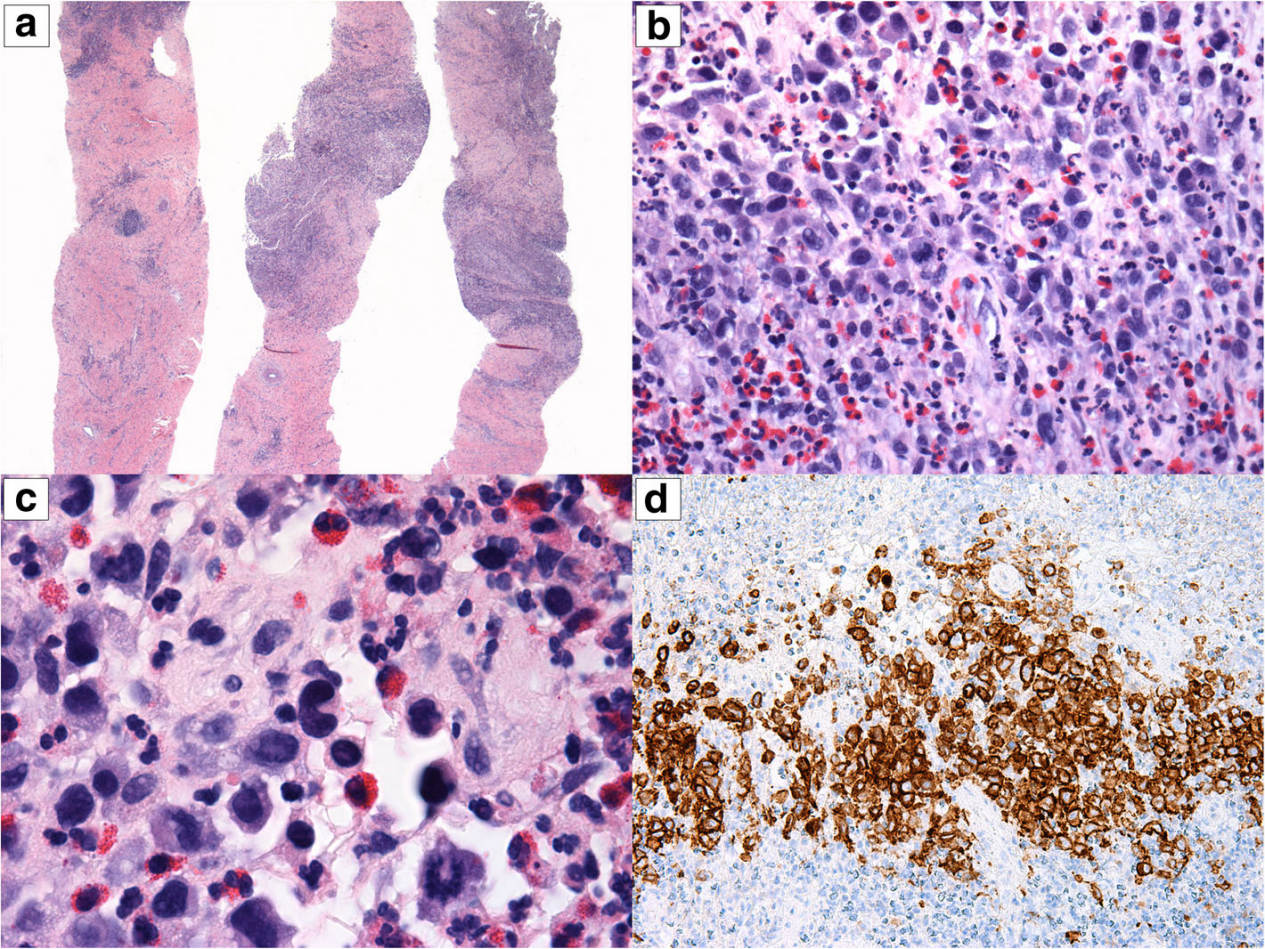
Breast implant-associated large cell lymphoma on core-needle biopsy. **a** Fragments of fibrous tissue exhibiting dense foci of lymphoid cells (H&E, 20×). **b** These foci are constituted by large atypical lymphoid cells interspersed with granulocytes, especially eosinophils (H&E, 400×). **c** Some of the large cells show kidney-shaped nuclei, the so-called “hallmark cells” (center of the field) (H&E, 1000×). **d** Strong membranous expression of CD30 in the neoplastic cells (200×)

Histological examination (H&E) showed infiltration of the capsule’s fibrous tissue by a dense population of granulocytes, especially eosinophils, interspersed with large atypical lymphoid cells. The latter showed moderate pleomorphism, high nuclear to cytoplasmic ratio and easily found mitotic figures. In addition, some of them exhibited eccentric, kidney-shaped nuclei, with homogeneous eosinophilic cytoplasm (“hallmark cells”) (Fig. 2).

**Fig. 3:**
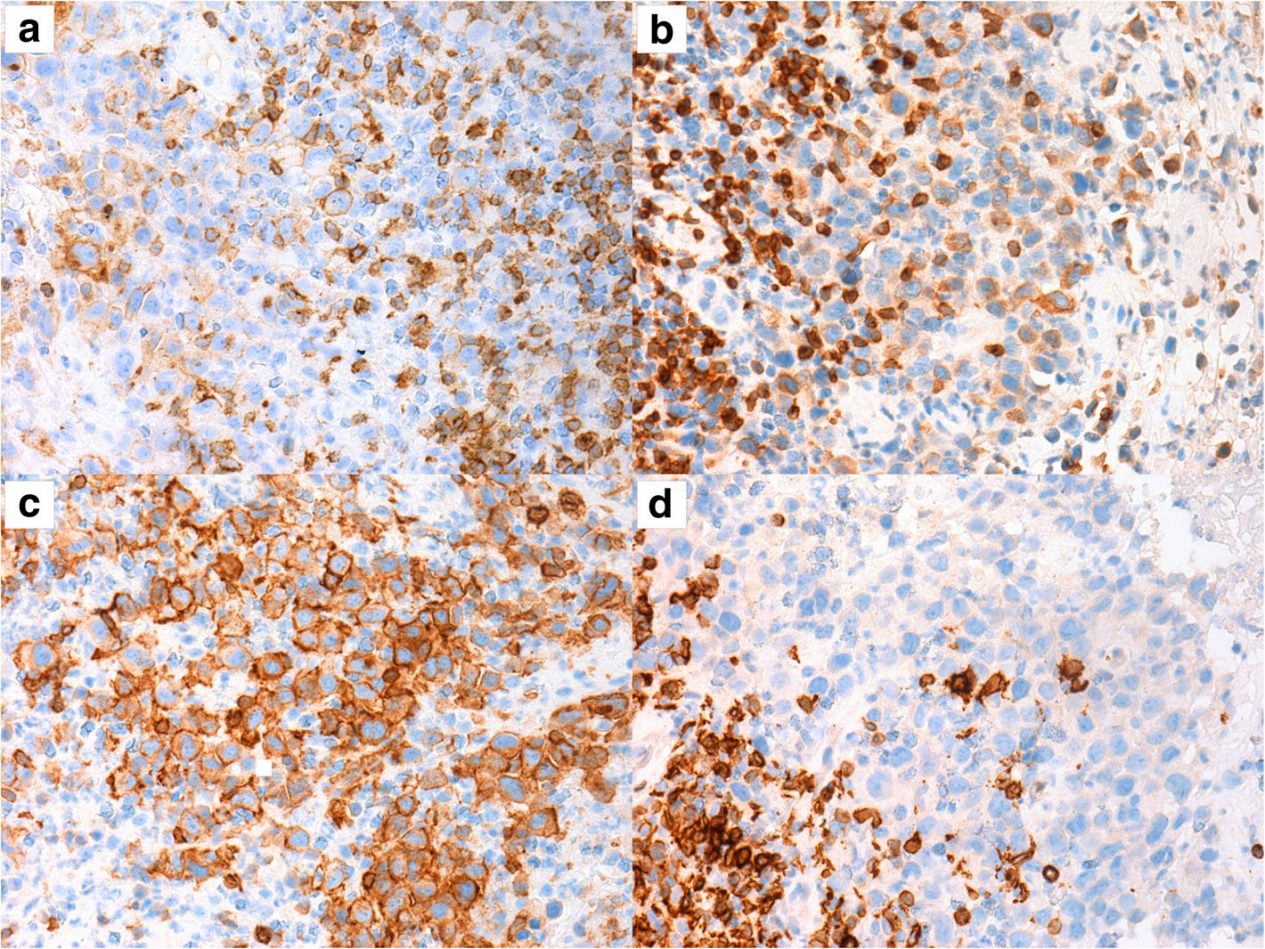
Immunohistochemical panel performed on the biopsy material. The large neoplastic cells showed varied degrees of positivity for CD2 (**a**), CD3 (**b**), CD5 (**c**), and CD7 (**d**) (400×). Notice that small reactive lymphocytes function as positive control. The marker with the highest number of positive neoplastic cells was CD5, whereas that with the highest deletion was CD7

**Fig. 4:**
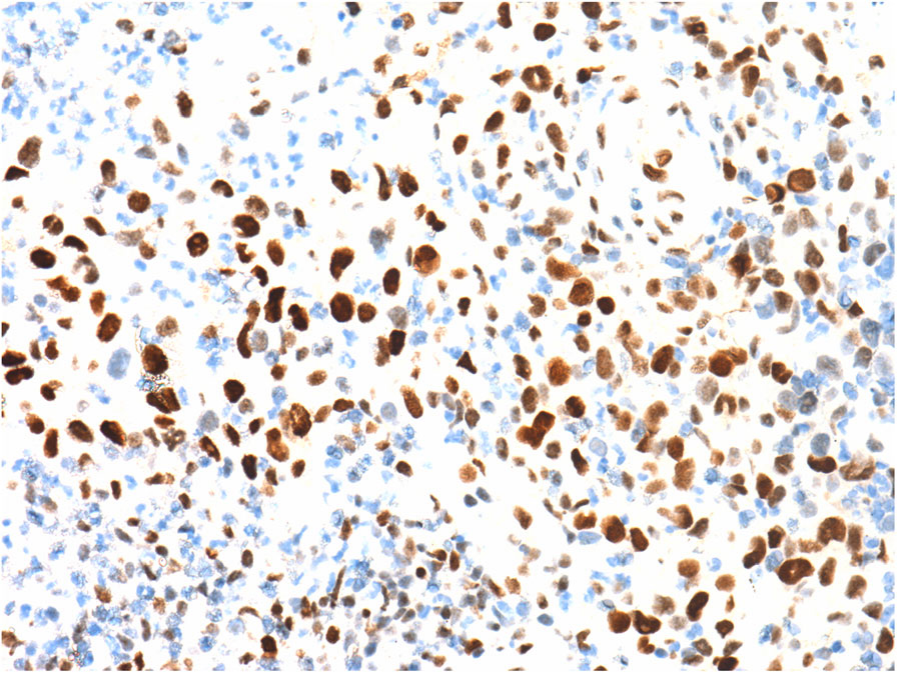
Immunohistochemical expression of p53 protein in the nuclei of neoplastic cells (400×)

The atypical cells showed strong and diffuse expression of CD30 on immunohistochemistry (IHC) (Fig. 2d). CD2, CD3, CD4 and CD5 were at least focally positive in the malignant cells. CD5 was expressed by the highest number of tumor cells, whereas CD7 was more extensively deleted (Fig. 3). CD8 was negative. P53 protein was extensively expressed by the atypical cells (Fig. 4) and Ki-67 proliferation index was high (80%). There was no expression of AE1/AE3, CD20, PAX5, CD68, ALK and TIA-1 on the atypical cells. The diagnosis of BIA-ALCL was rendered. One month later, the patient underwent right modified radical mastectomy with breast implant excision and axillary region dissection. Microscopic evaluation of the resection specimen showed the implant capsule infiltrated by the neoplasm. All dissected lymph nodes were uninvolved by atypical cells. At the time of this manuscript’s submission the patient was about to start systemic therapy.

## Discussion and conclusions

In the present report, a further case of BIA-ALCL is added. This case presents similarities with previous reports, but also some particularities, which should be stressed to make the diagnosis the earliest possible. The most distinct feature is that this is the second report of association of BIA-ALCL with Li-FRAUMENI syndrome.

As illustrated by our patient, symptoms of this type of lymphoma often include breast swelling and skin rash [7]. Most commonly, the tumor exhibits recurrent periprosthetic effusions, and less frequently, mass forming lesions years after implant surgery [7].

Current imaging studies appear suboptimal in the detection of BIA-ALCL, as these neoplasms seem to show no specific signs on imaging. Nonetheless, ultrasound and MRI have proved to be the most sensitive methods in detecting implant-associated effusions. On the other hand, the sensitivity in detecting mass-forming cases have varied among imaging modalities [8].

Cytological examination resulted negative in our patient. Recent consensus guidelines recommend fine-needle aspiration (FNA) of late symptomatic peri-prosthetic collections post implant surgery [9]. Cytomorphology of BIA-ALCL samples are usually highly cellular, with large atypical lymphoid cells within fibrinous or inflammatory background. Cell block preparations can be of great help, as they are more adequate for immunocytochemical studies [10]. Mass-forming lesions, however, should be preferentially submitted to core needle biopsy [9].

Laurent et al. [11] described two histological aspects of these tumors: (1) in situ BIA-ALCL, represented by a neoplastic lymphoid proliferation confined to the implant capsule, mostly associated with the effusion cases; and (2) infiltrative BIA-ALCL, as in the present case, with the tumor cells clearly infiltrating the capsule and/or adjacent tissues, mostly related to mass-forming lesions. Both clinicopathological patterns may coexist in the same patient. The neoplastic cells are usually large and anaplastic, with some “hallmark cells”; a background of eosinophils can be appreciated [7]. Immunophenotypically, the malignant cells show diffuse expression of CD30, frequent positivity for one or more T-cell and/or cytotoxic associated markers, and EMA. ALK staining is consistently negative [1, 7, 11].

In the past decades, the development of clonality studies have done major contributions to the diagnosis and management of hematological neoplasms. These studies are able to detect common gene rearrangements in the malignant lymphoid cells, usually not present in benign lymphoid proliferations [12]. As BIA-ALCL can often be misdiagnosed as a benign inflammatory process, clonality analyses could be of great diagnostic utility in such cases. Molecular studies on BIA-ALCL have evidenced T cell receptor gene rearrangements in the majority of cases and few cases with polyclonal rearrangements in immunoglobulin chain genes [10, 11]. However, this technique is still not available at our laboratories. Therefore, we had to base our diagnosis on morphological and immunohistochemical grounds. Aberrant phenotype supports the neoplastic nature of the atypical cell infiltrate: strong, diffuse expression of CD30, and partial deletion of pan-T markers (see Fig. 3). Strong expression of p53 protein also favor the neoplastic nature of atypical cells (Fig. 4).

The precise pathogenic mechanisms of this type of lymphoma are still to be established. Chronic bacterial antigen stimulation or implant characteristics, as texture and chemical composition, may play a role in tumorigenesis, as it occurs in other inflammation related lymphomas, such as primary cutaneous CD30+ lymphomas, which might be associated to chronic atopic eczema [13, 14].

In our case, the patient was previously diagnosed with Li-FRAUMENI syndrome, an inherited autosomal dominant disorder, with elevated cancer predisposition due to abnormalities in the p53 tumor suppressor gene (TP53) [15]. It is noteworthy that not all tumors positively stained for p53 on IHC are associated with mutations in this gene and the diagnosis of LFS should be based on genetic testing for clinically selected individuals [15, 16]. In ALCL, p53 expression was found in the majority of cases, but this did not necessarily correlated with evidence of gene mutation [17, 18]. In the present case, LFS and TP53 abnormalities might represent additional underlying conditions for the development of BIA-ALCL, a hypothesis also raised in the first report on this type of association [19]. In that report, the authors discussed the role of previous chemo- and radiation therapy in lymphomagenesis. However, in our case, no previous treatment for breast cancer had been given, except for tumor resection. Therefore, pathogenesis involves more likely the genetic syndrome, and/or antigenic stimulation. The rarity of such association hinders a more precise explanation on the pathogenesis of ALCL in our patient.

Delay in diagnosis has been reported in up to 40% of cases [5]. This seems to be due to the lack of awareness for this entity both by patients and physicians, who mainly consider the diagnosis of inflammation or scar. As expected, postponement may influence therapy and outcome [7–9].

In summary, we report herein a remarkably rare case of BIA-ALCL arising in a patient with Li-FRAUMENI syndrome after bilateral mastectomy for breast carcinoma, with implant reconstruction. Although increasing data have been published on this entity in recent years, patients and health professionals should be aware of this diagnosis in order to avoid late diagnoses.

## Abbreviations

ALCL: Anaplastic large cell lymphoma kinase
ALCL, ALK: ALK-negative anaplastic large cell lymphomas
ALK: Anaplastic large cell lymphoma kinase
BIA-ALCL: Breast implant-associated anaplastic large cell lymphoma
FNA: Fine-needle aspiration
H&E: Hematoxylin and eosin
IHC: Immunohistochemistry
LFS: Li-FRAUMENI syndrome
MRI: Magnetic resonance imaging
WHO: World Health Organization
